# Exploring central star variability of planetary nebulae using gaia photometry

**DOI:** 10.1038/s41598-026-42163-1

**Published:** 2026-03-25

**Authors:** M. A. NegmEldin, A. Ali, G. M. Hamed, A. Essam

**Affiliations:** 1 Astronomy, Space Science & Meteorology Department, Faculty of Science, Cairo University, Giza, 12613 Egypt; 2 Stellar Astronomy Lab, Astronomy Department, National Research Institute of Astronomy and Geophysics, 11421 Helwan, Cairo, Egypt

**Keywords:** Planetary nebulae, General, Stars, Binaries, Close, Stars, Variables, General, Techniques, Photometric, Gaia DR3, Astronomy and planetary science, Optics and photonics

## Abstract

This study presents an analysis of the variability of 81 central stars of planetary nebulae (CSPNe) are identified as variable in *Gaia* Data Release 3 (DR3). By combining *Gaia* time-series photometry with data from the Optical Gravitational Lensing Experiment (*OGLE*), the Transiting Exoplanet Survey Satellite (*TESS*), and the *Kepler*, we characterise their variability and identifed 17 new periodic systems. Among these, 13 are short-period variable: light-curve modelling reveals one eclipsing binary, two systems with combined eclipsing and ellipsoidal modulation, one with irradiation effect, two with irradiation/ellipsoidal effects and seven with uncommon variability. A further five systems exhibit long-period variability. Notably, the central star (CS) of PN Al 2-R displays dual periodicity–a short period of 0.99 days and a long period of 501.4 days–and is consequently included in both the short- and long-period categories. The host nebulae of the new short-period binaries are predominantly bipolar or elliptical, strengthening the link between binarity and asymmetric planetary nebula shaping. Furthermore, we provide the first systematic confirmation of binarity in 15 previously known variable CSPNe using Gaia data, with one further confirmation from *TESS*. For our variability-selected sample, we derive a short-period binary fraction of approximately 48%, exceeding values reported for non-selected samples and highlighting the impact of selection effects. Furthermore, the bipolar/elliptical fraction for wide-binary central stars (57–70%) remains lower than that for close binaries, reinforcing the stronger shaping influence of close binary interactions.

## Introduction

The Interacting Stellar Winds (ISW) model^1^ describes planetary nebula (PN) formation as the interaction between a slow, dense asymptotic giant branch (AGB) wind ($\sim$ 10 km/s) and a subsequent fast, tenuous wind ($\sim$ 1000 km/s), which produces the nebular shell. The ISW model provides a satisfactory description of spherical planetary nebulae but is inadequate for explaining non-spherical morphologies. To overcome this limitation, the Generalized ISW (GISW)^2–4^ model was formulated, featuring an axially symmetric AGB wind characterized by enhanced equatorial density. This asymmetry channels the fast wind along the poles, yielding a range of morphologies: mild equatorial contrasts produce elliptical nebulae, while strong contrasts generate bipolar “butterfly” nebulae. The GISW model therefore accounts for the observed continuum of PN structures.

Single-star evolution alone cannot reproduce the full diversity of morphologies–including elliptical, bipolar, and multipolar forms–or smaller-scale features such as jets and discs^3,5^. These complexities are shaped by various factors, including magnetic fields, binary central stars (BCSs), common-envelope (CE) evolution, and even interactions with massive planets^6^. Among these, binarity has emerged as a crucial factor in shaping PNe. Evidence indicates that many systems undergo CE interactions, where mass transfer during the AGB phase drives the formation of equatorial discs and accretion-driven jets, further enriching PN morphology^7^.

The main photometric signatures of close binarity are eclipses, ellipsoidal modulation, and irradiation effects. Eclipses occur when the orbital configuration allows one star to pass in front of the other, with visibility depending on inclination, stellar sizes, and separation. Ellipsoidal modulation arises in close systems where stars are tidally distorted, producing quasi-sinusoidal variability. Irradiation effects occur when stellar hemispheres facing the companion are heated, creating a brightness contrast that varies with orbital phase. Irradiated systems show a single minimum per orbit, whereas ellipsoidal variables exhibit two; in eclipsing irradiated binaries, the eclipses align with the maxima and minima of the irradiation-induced variation^7^.

Periodic variability in CSPNe, however, can also result from other mechanisms. Pulsations, caused by expansions and contractions of the stellar envelope, and rotational modulation, produced by star spots on a cooler companion, are notable examples^8–10^. Rotational acceleration of companions may occur when material lost during the AGB phase is accreted, transferring angular momentum^11^.

Over the past two decades, photometric surveys have identified more than one hundred binary CSs^7^. The *OGLE* survey provided the first large sample^12,13^, followed by detections with *TESS*^14,15^ and *Kepler*/K2^16^. More recently, *ZTF* has significantly advanced the field with the identification of 94 variable CSPNe (including six new periodic systems) and the reporting of 39 periodic variables (19 of them new)^11,17^.

Paper I^18^ reported a set of variable central stars from *Gaia* DR3 comprising 26 symbiotic stars and 81 CSPNe (corrected from an initial count of 82 due to duplication of Tc 1).

The present study builds on Paper I by systematically analysing the variability of the CSPNe sample using *Gaia* time-series data. The aims are to expand the catalogue of close binaries through new discoveries and confirmations, thereby strengthening the statistical foundation for studying their properties, and to test the correlation between close binarity and asymmetrical nebular morphologies.

This article is organised as follows: Section Materials and methods describes the sample, data acquisition, and methodology. Section Results and discussion presents the results and discussion. Section Conclusions summarises the conclusions.

## Materials and methods

### Initial sample

Our analysis builds upon the sample of 81 variable CSPNe candidates identified in Paper I from *Gaia* DR3. This initial sample (Sample 1) contains 32 known close binaries (revised upward from the 24 initially reported in Paper I), 4 known pulsators (NGC 1501, NGC 2371, PN K 1–16^8^, and PN Lo 4^19^), and two objects–PN M3-2^20^ and PN SuWt 2^21^–that are now considered chance alignments rather than true CSPNe, leaving 43 new variable candidates. Recent observations from *TESS* photometry have confirmed the close binary nature of PG 1034+001^15^ (Note that PG 1034+001 was not part of our final working sample and thus does not appear in Groups I or II). Additionally, independent analyses of Gaia DR3 data have identified nine of these candidates as long-period variables (LPVs)^22^ and approximately 20 as eclipsing binary candidates^23^. While these independent Gaia DR3 identifications offer a preliminary context for CSPN variability, our primary analysis is restricted to Sample 2, where the data are sufficient for robust period determination.

### Final working sample (sample 2)

Sample 2 was defined in a two-step process. First, we compiled the complete Gaia DR3 time-series photometry for each central star in Sample 1. Second, we selected only those objects whose data were sufficient to generate a clear, interpretable light curve suitable for reliable period analysis, thereby forming the final working set. Central stars with data too limited for this purpose were omitted. For targets where Gaia DR3 data alone were inadequate, we incorporated photometry from *TESS*, *OGLE*, and *Kepler* to ensure comprehensive coverage. The properties of Sample 2 objects are listed in Table 1, which includes the PN common name, coordinates, *Gaia* identifier, *G* magnitude, PN status, and PN morphological class.

**Table 1:** Fundamental properties of sample 2 objects.

PN	l	b	RA	dec	Gaia DR3	G	Status	PN
name	(deg.)	(deg.)	(deg.)	(deg.)	designation	magintude		morphology
PPA J1800-2904	1.5170	−2.8505	270.0934	−29.0776	4062356711999251328	18.15	T	S
PN ShWi 7	1.7958	−3.8754	271.2730	−29.3376	4050366645122261504	17.87	T	B
PN Hf 2-2	5.1379	−8.9029	278.1287	−28.7223	4048497024309080064	17.16	T	Ems
PN H 2–22	6.3421	3.3297	266.8914	−21.7898	4117062676912301184	18.47	T	B
PN A66 41	9.6620	10.5141	262.2584	−15.2179	4136835641106850432	16.24	T	Bas
PN Sa 3–111	14.2721	4.2132	270.2781	−14.5059	4147061232357104384	16.97	T	S
PN G054.5+01.8	54.5879	1.8518	291.3954	20.0596	4515887189511585792	18.64	L	E
PN A66 46	55.4133	16.0310	277.8275	26.9367	4585381817643702528	14.96	T	Eas
IRAS 19461+2419	60.9866	−0.5698	297.0595	24.4575	2020643612977496704	18.56	T	S
ETHOS 1	68.0976	10.9864	289.1312	36.1632	2050526964622031744	17.20	T	Bmps
MWP 1	80.3556	−10.4093	319.2845	34.2077	1855295171732158080	13.02	T	Baps
PN M 1–77	89.3840	−2.2664	319.7807	46.3131	1971995510535755648	11.94	T	Sm
PN HFG 1	136.3809	5.5526	45.9460	64.9098	468033345145186816	13.99	T	Eamrs
LTNF 1	144.8107	65.8463	179.4369	48.9384	786919754746647424	15.10	T	Bas
PHR J0650+0013	212.6413	−0.0651	102.6690	0.2290	3113542949606809088	15.25	T	Bmps
IC 2165	221.3240	−12.3943	95.4283	−12.9872	2999839084924027776	17.50	T	Emrs
PN K 1–2	253.5772	10.7796	134.4415	−28.9602	5647809392112960000	17.01	T	Baps
PN Lo 4	274.3093	9.1120	151.4407	−44.3593	5414927915911816704	16.59	T	Ears
Wray 16–55	277.6238	−1.7300	144.8912	−54.8819	5308685822467307008	12.17	T	S
PN K 1–22	283.6719	25.3141	171.6824	−34.3698	5399388964749811456	16.71	T	Ears
PN DS 1	283.9032	9.7258	163.6689	−48.7841	5362804330246457344	12.14	T	Ims
PN G305.9-01.2.2	305.9325	−1.2698	199.7114	−63.9818	5859151160662602752	19.32	T	B
PN Sp 1	329.0787	1.9571	237.9206	−51.5246	5982072132545824128	13.73	T	Ramrs
PN HaTr 7	332.5068	−16.9108	268.5393	−60.8327	5911656865276078080	14.83	T	Eas
PN HaTr 4	335.2517	−3.6205	251.2508	−51.2059	5937103069115240192	16.78	T	B
NGC 6026	341.6049	13.7042	240.3380	−34.5433	6011169161583903488	13.13	T	Eas
IC 1266 (TC 1)	345.2374	−8.8350	266.3970	−46.0899	5954912374289120896	11.27	T	Rars
PPA J1747-3435	355.3275	−3.2125	266.7847	−34.5953	4041711044735017856	18.78	T	Es
PN M 1–27	356.5312	−2.3941	266.6895	−33.1431	4053955824662571648	13.95	T	R
PN M 4-4	357.0340	2.4401	262.2095	−30.1292	4058620300987916160	16.53	T	Ear
PN Al 2-R	358.7512	−2.7585	268.4019	−31.4239	4055678213978728320	15.42	T	B

Note: Status codes: “T” = True CSPN, “L” = Likely CSPN. PN Morphology codes: B = Bipolar, E = Elliptical, R = Round, S = Stellar/unresolved. Lowercase letters denote finer structural features: s = multiple shells, m = point symmetry, p = ring structure, r = asymmetry.).

### Data sources and period analysis

Time-series photometry was primarily obtained from the *Gaia* mission, which provides data in its G (350–1000 nm), BP (330–680 nm), and RP (640–1000 nm) passbands^24^. These data were complemented by observations from the *TESS*, *OGLE*, and *Kepler* surveys. The *TESS* observations used a wide red filter (600–1000 nm), while *OGLE* employed B, V, and I filters, with the I-band serving as the principal filter in most *OGLE* observations, particularly for the Galactic disk and bulge^25^. The *Kepler* mission used a broad visible-light bandpass (420–900 nm).

The determination of periodic signals was conducted with the VStar software package. This tool employed the Data-Compensated Discrete Fourier Transform algorithm to detect significant periods in the light curves. To validate these findings, we additionally employed the Lomb-Scargle periodogram method^26^.

## Results and discussion

We present results for Sample 2, divided into Group I (17 newly identified periodic variables) and Group II (15 previously known binaries reanalysed with *Gaia*).

### New periodic variables (Group I)

We discovered 17 new periodic variable CSPNe, comprising 12 short-period binaries, 4 long-period systems, and one system with both short- and long-period variability. Our analysis confirms periodic variability in eight objects using *Gaia* data, in five using *TESS*, in three using *OGLE*, and in one using *Kepler*. To assess the potential for photometric contamination from nearby sources in the *TESS* and Kepler data, we extracted the CROWDSAP factor from the FITS file headers. The analysis reveals varying levels of contamination: M 4-4 has a low level ($\sim 10\%$), MWP 1 is moderate ($\sim 50\%$), and Lo 4 is severe ($\sim 90\%$). For PN M 1–77, PN M 1–27, and PN Tc 1, the CROWDSAP factor is unavailable. Consequently, the periods derived for these three objects should be considered less certain. The derived periods of five objects in this group are uncertain (marked with an asterisk, “*” in Table 1), primarily due to limitations in *Gaia* DR3 photometry.

**Fig. 1:**
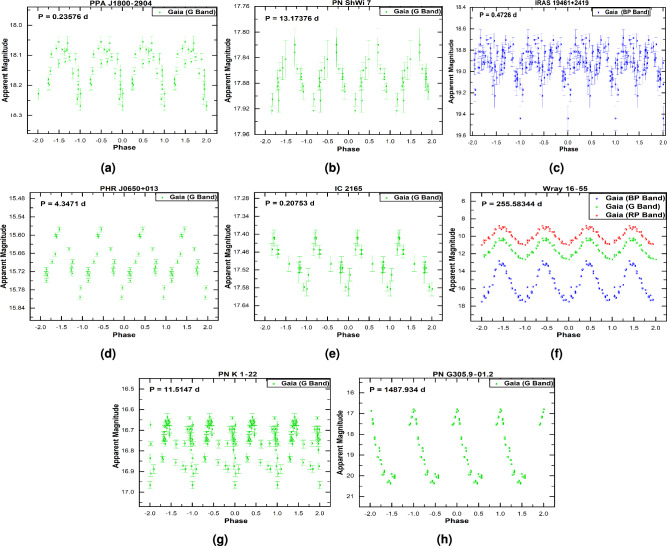
The phase-folded light curves of Group I objects obtained from *Gaia* photometry.

**Fig. 2:**
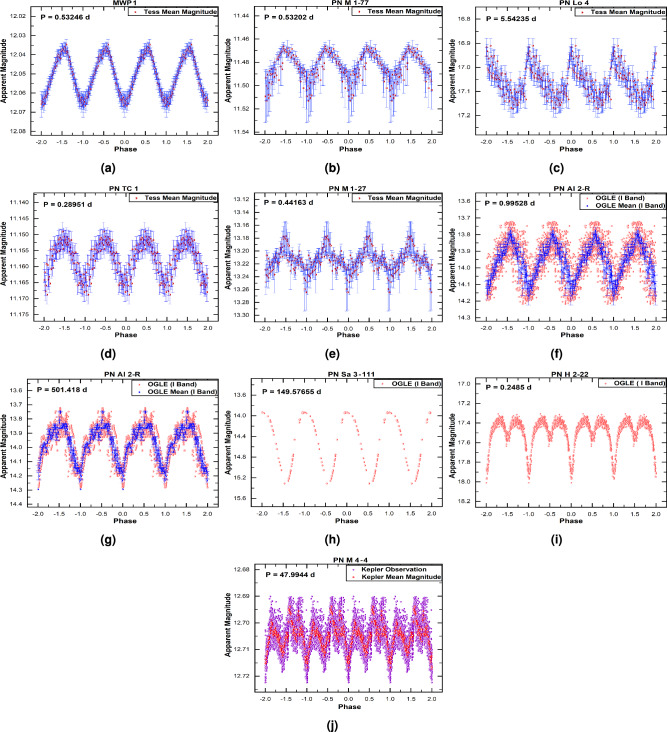
The phase-folded light curves of Group I objects obtained from *TESS*, *OGLE*, and *Kepler* photometry.

The Phase-folded light curves of this group, derived from *Gaia* data are shown in Fig. 1, and those derived from *TESS*, *OGLE*, and *Kepler* are presented in Fig. 2. Additionally, Table 2 summarises the characteristics of this Group, listing the photometric data source, ephemeris equation, amplitude variation, proposed variability type, and nebular morphology. In terms of nebular morphology, the sample includes seven bipolars (B type), three ellipticals (E type), two round (R type) nebulae, and five unresolved “quasi-stellar” (S type) objects.

**Table 2:** Properties of newly identified periodic variable CSPNe (Group I). Ephemeris: HJD (Min.I) = Epoch + Period $\times$ E. Uncertain periods are marked with (*). Abbreviations are as follows: Binary Type – Ecl (Eclipsing), Irr (Irradiation), Ell (Ellipsoidal); Amplitude of Variation – Amp. Var.

PN Name	Data	Ephemeris equation	Amp. Var.	Binary	PN
	Source	Epoch (HJD)	(mag.)	type	Morphology
**Short-period variables**					
PPA J1800-2904	*Gaia*	2457320.6415+0.235756605$\times$E	0.20	Ecl+Ell	*S*
PN ShWi 7${^*}$	*Gaia*	2457455.75924+13.173759$\times$E	0.11	Irr/Ell	*B*
IRAS 19461+2419	*Gaia*	2457759.69198+0.4726$\times$E	0.77	Ecl+Ell	*S*
PHR J0650+0013${^*}$	*Gaia*	2457099.49454+4.347041$\times$E	0.28	Irr/Ell	*Bmps*
IC 2165${^*}$	*Gaia*	2457653.92868+0.20753$\times$E	0.23	?	*Emrs*
PN K 1–22 ${^*}$	*Gaia*	2457395.82103+11.5147$\times$E	0.33	?	*Ears*
MWP 1	*TESS*	2458712.19656+0.532458$\times$E	0.032	?	*Baps*
PN M 1–77	*TESS*	2458717.00927+0.532017$\times$E	0.045	?	*S*
PN Lo 4	*TESS*	2459298.26561+5.54235$\times$E	0.26	?	*Ears*
PN TC 1	*TESS*	2459376.11313+0.289507$\times$E	0.021	?	*Rars*
PN M 1–27	*TESS*	2459370.88422+0.441629$\times$E	0.086	?	*R*
PN H 2–22	*OGLE*	2456142.53771+0.24850134$\times$E	0.71	Ecl	*B*
PN Al 2-R	*OGLE*	24543812.89822+0.9952767$\times$E	0.0.44	Irr	*B*
**Long-period variables**					
Wray 16–55	*Gaia*	2457777.88523+255.58344$\times$E	2.43	LPV	*S*
PN G305.9-01.2.2${^*}$	*Gaia*	2457049.69868+1487.934$\times$E	4.7	LPV	*B*
PN M 4-4	*Kepler*	2457704.92268+47.9944$\times$E	0.032	LPV	*Ear*
PN Sa 3–111	*OGLE*	2457953.54793+149.57655$\times$E	1.40	LPV	*S*
PN Al 2-R	*OGLE*	2454576.87537+501.418$\times$E	0.56	LPV	*B*

**Note:** The central star of PN Al 2-R exhibits two distinct periods: a short one of 0.99 days and a long one of 501.4 days.

Light-curve analysis attributes the variability in the short-period group to eclipsing phenomena (1 system), a combination of eclipsing and ellipsoidal modulation (2 systems), irradiation effect (1 system), and irradiation/ellipsoidal effects (2 systems), confirming their close binary nature. The variability type for the remaining seven systems remains undefined. Most systems exhibit low-amplitude variations (0.02–0.4 mag), and their period distribution–which peaks below one day–is consistent with the established peak for close binary systems^11,16,17^.

A key result is the morphological connection between the newly identified short-period binaries and their host nebulae: excluding objects with unresolved classifications (S type), nearly 80% (8 out of 10) display B and E morphologies. This provides strong observational support for the hypothesis that binary interactions are a major driver of asymmetry in PNe. In contrast, a control sample from the HASH catalogue shows that only about 66% (1,840 of 2,790) of true PNe display such asymmetric morphologies. This result is interesting because it matches what binary interaction models predict, but we cannot draw strong statistical conclusions from such a small sample (10 objects).

Furthermore, to examine the roles of close- and wide-binary central stars (CSs) in shaping planetary nebulae, we consider 14 high probable wide-binary CSs with well-defined morphologies^27^. Eight show B/E forms and six are R, giving a B/E fraction of 57%. Adding systems from an earlier study^28^ raises this to 70%, though still below the fraction for close binaries. Given the small sample size, this comparison should be viewed cautiously.

The upcoming *Gaia* DR4, with a doubled time baseline, will be crucial for confirming the uncertain systems and discovering more variables with even lower amplitudes and longer periods, further refining our understanding of the binary CSPN population.

**Fig. 3:**
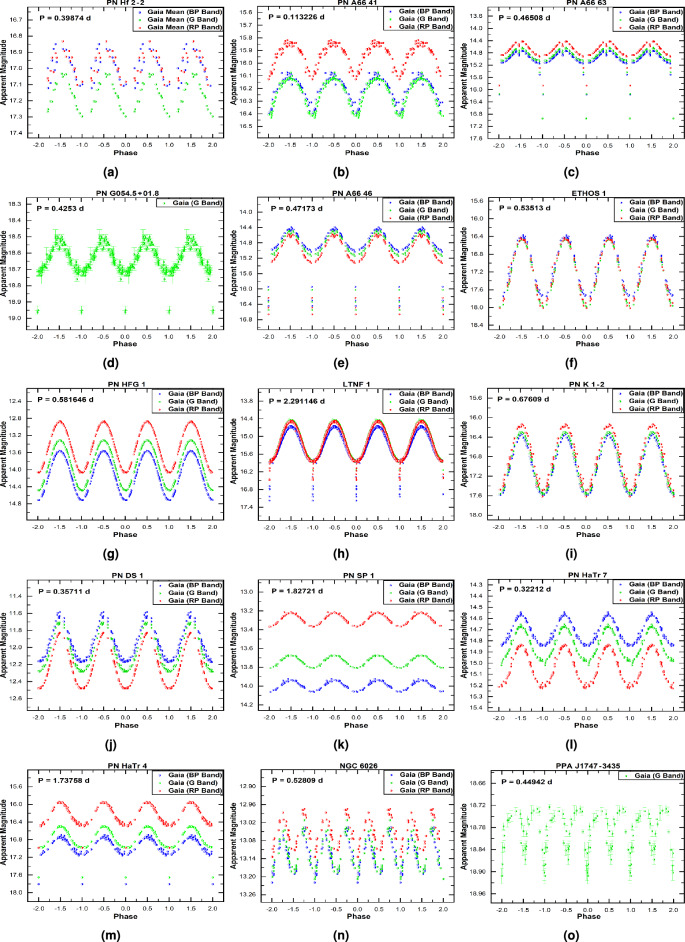
The phase-folded light curves for Group II objects, created from *Gaia* DR3 photometric data.

We briefly discuss some of the newly identified periodic variables in Group I:

- **PN ShWi 7 and PHR J0650+0013:** Both systems exhibit clear, periodic light curves with orbital periods of $13.17\ \textrm{d}$ (ShWi 7) and $4.35\ \textrm{d}$ (PHR J0650+0013). Their light curves are asymmetric and show no evidence of eclipses or secondary minima, confirming their binary nature but making the precise physical origin of the variability ambiguous from photometry alone. While the smooth, single-wave modulation could be consistent with effects such as irradiation or ellipsoidal variation, we cannot securely distinguish between these (or other) mechanisms with the current dataset. Therefore, in Table 2 we classify both as irradiation/ellipsoidal candidates, reflecting this ambiguity and the need for future spectroscopic or multicolor photometric follow-up to determine the precise nature of the variability.
- **PPA J1800-2904 and IRAS 19461+2419:** Analysis of *Gaia* DR3 photometry reveals that both systems host close binary CSs with remarkably short orbital periods of 0.236 d and 0.473 d, respectively. The light curve of PPA J1800-2904 (Fig. 1, panel a) indicates an eclipsing and ellipsoidal binary system–a classification consistent with its inclusion in the *Gaia* catalogue of eclipsing binary candidates^23^. The light curve of IRAS 19461+2419 (Fig. 1, panel c) shows also characteristics of eclipsing and ellipsoidal binary system. Given the established correlation between close binarity and asymmetric nebular formation^29^, we predict that both PNe possess bipolar or highly elliptical morphologies. Their current point-like appearance in the HASH catalogue is attributed to resolution limitations in existing observations. This hypothesis can be tested through high-resolution imaging with facilities such as the *VLT* or *HST*, following methodologies successfully demonstrated for similar systems^30^.
- **PN H 2–22:** We confirm PN H 2–22 as an eclipsing binary system with a robust period of 0.2485 d. While previous studies suggested tentative classifications of possible shorter periods, our high-quality *OGLE* light curve reveals a clear eclipse signature. One study^11^ reported a scattered 0.1421 d signal that could be an alias, especially for a doubly eclipsing system, while another^17^ identified a most probable period of 0.1242 d but described the light curve as noisy. Our data firmly establish the 0.2485 d period, with the previously reported values likely representing half-period aliases. The *OGLE* I-band morphology, resembling that of the known binary central star PTB 26, together with its known bipolar nebular structure, provides consistent evidence for a binary nucleus.
- **MWP 1 and PN Lo 4:** MWP 1 has been reported as a hot subdwarf candidate^31^, a pulsating hydrogen-deficient white dwarf^8^ and as a pulsating hot subdwarf B star^32^. PN Lo 4, in addition to being a hot subdwarf^31^, is a multiperiodic nonradial pulsator^33^ and exhibits a spectacular transient mass-loss event^19^. It is also catalogued as a variable hot subdwarf^34^. The light curves of both MWP 1 and PN Lo 4 exhibit low-amplitude variability with profiles that are difficult to associate with binary systems. In both cases, analysis of the Target Pixel Files (TPF) with the TESS-Localize package confirms that the detected variability is genuine. For PN Lo 4, which suffers from severe contamination, the variability amplitude of the background and surrounding stars is significantly lower than that of its CS. Similarly, for MWP 1, which has moderate contamination, the background shows very low variability compared to the significant signal from the CS. We are therefore confident that the observed variability for both objects is intrinsic to the stars themselves and not a background artifact.
- **PN M1-77, PN M1-27, and PN Tc 1:** The light curves of these CSs reveal variable systems with orbital periods of 0.53 d, 0.44 d, and 0.29 d, respectively. Photometric data for all three objects, obtained from the *TESS* database, indicate amplitudes less than 0.1 mag, with PN M1-77 showing $\sim$0.05 mag, PN M1-27 $\sim$0.09 mag and PN Tc 1 $\sim$0.02mag. Although irradiation effects could explain the variability of PN Tc 1, this mechanism is less likely for PN M1-77 and PN M1-27. Despite their short orbital periods and sinusoidal-like light curves, the observed modulations lack the distinct symmetry characteristic of irradiation–as clearly seen, for comparison, in the light curves of PN DS 1 and PN HaTr 7 (Fig. 3, panels j and l, respectively). In the case of PN Tc 1, the variability could alternatively be interpreted as ellipsoidal modulation or even a contact eclipsing binary. As noted above, the potential contamination from nearby stars in the *TESS* data could not be assessed for these three objects. Therefore, the results of the light curve analysis–specifically, the amplitude variation and period values–should be treated with caution. Radial-velocity variability in PN M1-77 suggests it is very likely a spectroscopic binary system^35^, but spectroscopic follow-up will be essential to confirm the nature of all three systems. Morphologically, M1-27 and Tc 1 appear round, while M1-77 is classified as S-type. Such shapes might seem inconsistent with close binarity; however, a bipolar nebula viewed at a moderate inclination (i.e., not fully edge-on) can project a roughly circular or elliptical outline on the sky. In particular, a detailed 3D morpho-kinematical model of Tc 1^36^ reproduces its [S II] emission as a slightly elongated spheroid with an equatorial density enhancement, consistent with a bipolar structure seen at a relatively low inclination. For M1-77, the detection of radial-velocity variations implies that the binary orbit is not face-on; hence, if the nebula is indeed bipolar, its symmetry axis is likely not aligned exactly with the line of sight, allowing both a non-zero orbital inclination and a nebular appearance that is not overtly bipolar in projection.
- **IC 2165 and PN K 1–22:** *Gaia* DR3 reveals atypical variability in both systems that is inconsistent with binarity. The light curve of IC 2165 (period $\sim$0.208 d) shows a slight, sawtooth-like pattern (Fig. 1, panel e), similar to that observed in PN Lo 4 (Fig. 2, panel c). In contrast, PN K 1–22 exhibits a distinct and uncommon light-curve morphology; the associated timescale ($\sim$11.5 d) is uncertain and may reflect an alias rather than a well-defined periodicity.
- **PN Al 2-R:** PN Al 2-R is a particularly complex system, for which we detect two distinct periods: a short period of 0.999 d indicative of an irradiated close binary (Fig. 2, panel f), and a long period of 501.42 d (Fig. 2, panel g). This suggests the CS is both a binary and a pulsator, offering a unique opportunity to study these interacting phenomena. In the *OGLE*-III catalogue of Galactic bulge long-period variables, this object is listed with three different periods: 58.4 d, 225.3 d, and 514.3 d^37^. The bipolar morphology of its host nebula confirms the past influence of the close binary interaction.
- **PN M 4-4, Wray 16–55, etc. (LPVs):** The classification of five systems (Wray 16–55, PN G305.9-01.2.2, PN M 4-4, PN Sa 3–111, and PN Al 2-R) as LPVs is consistent with previous studies^22,38^. The light curves of Wray 16–55, PN G305.9-01.2.2, and PN Sa 3–111 exhibit the characteristics of pulsating Mira variables, with large amplitudes and long, regular periods typically ranging from 80 to over 1000 d. A literature review confirms that both Wray 16–55 and Sa 3–111 have previously been classified as Mira variables, with periods of 256.9 d and 159.99 d, respectively^39^—consistent with our own period estimates. Their Mira-like behavior points toward a possible D-type symbiotic nature, i.e., a binary consisting of a pulsating AGB star (a Mira) and a white dwarf embedded in a dusty envelope. Although optical spectroscopy for definitive emission-line diagnostics is unavailable, we examined their infrared colors using the $J\text {-}H$ versus $H\text {-}K$ diagram^40^. All three objects fall outside the region occupied by planetary nebulae and instead overlap with the area of symbiotic stars, supporting a symbiotic interpretation.

### Group II CSPNe

We have performed the first homogeneous analysis of 15 known binary CSPNe using *Gaia* DR3 photometry. Periodic variability is recovered in nearly all systems across the three *Gaia* passbands (*G*, *BP*, and *RP*), with the exception of PNG 054.5+01.8 and PPA J1747−3435, where variability is detectable only in the *G* band. In addition, we determined the orbital period of NGC 6026 for the first time from *TESS* photometry. This object was previously confirmed as a close binary system with a period of 0.528086 d, with its light variations attributed to ellipsoidal distortion of the central star that nearly fills its Roche lobe, accompanied by a probable hot white dwarf companion^41^. The orbital period obtained from our *TESS* analysis (0.52833 d) agrees closely with the earlier determination.

**Table 3:** Properties of previously known binary CSPNe (Group II) analysed with *Gaia* and *TESS*.

PN Name	Data	Ephemeris equation	Amp. Var.	Binary	Known period
	Source	Epoch (HJD)	(mag.)	type	(days)
PN Hf 2-2	*Gaia*	2457866.09686+0.39873943$\times$E	0.36	Irr^45^	0.4^35^, 0.4^45^
PN A66 41	*Gaia*	2457451.93089+0.11322606$\times$E	0.35	Ell^45^	0.113^46^, 0.23^45^, 0.113^47^, 0.227^17^
PN A66 63	*Gaia*	2457380.39024+0.46507659$\times$ E	0.4	Ecl, Irr^45^	0.465^46^, 0.465^48^, 0.465^49^, 0.46^45^, 0.465^15^
PNG054.5+01.8	*Gaia*	2456996.34465+0.4253019$\times$E	0.25	Ecl, Irr^50^	0.425^50^
PN A66 46	*Gaia*	2457164.87258+0.4717275$\times$E	0.55	Ecl, Irr^45^	0.472^46^, 0.47^45^, 0.472^15^, 0.472^11^, 0.472^17^
ETHOS 1	*Gaia*	2457066.16423+0.5351272$\times$E	1.5	Irr^51^	0.53^51^; 0.535^52^, 0.54^11^, 0.535^13^
PN HFG 1	*Gaia*	2457893.93761+0.5816464$\times$E	1.2	Irr, Ell^45^	0.582^46^, 0.58^45^, 0.58^11^, 0.582^13^
LTNF 1	*Gaia*	2456912.36124+2.291146$\times$E	1.02	Ecl, Irr^45^	2.29^11^, 2.29^13^, 2.291^17^
PN K 1–2	Gaia	2457188.00705+0.67608676$\times$E	1.3	Irr^45^	0.676^46^, 0.676^53^, 0.68^45^
PN DS 1	*Gaia*	2457192.67226+0.357112$\times$E	0.58	Irr^45^	0.357^46^, 0.357^15^, 0.36^45^
PN SP 1	Gaia	2457677.40837+1.8272069$\times$E	0.15	Irr^45^	2.91^46^, 2.91^45^, 2.91^48^
PN HaTr 4	*Gaia*	2457586.46384+1.737583$\times$E	0.5	Irr^45^	1.71^46^, 1.74^45^,1.738^54^
PN HaTr 7	*Gaia*	2456986.52712+0.32212345$\times$E	0.55	Irr^55^	0.322^55^
NGC 6026	*Gaia*	2457302.70368+0.52808728$\times$E	0.20	Ell^45^	0.53^41^, 0.53^45^
PPA J1747-3435	*Gaia*	2457131.88368+0.4494214$\times$E	0.18	Ecl^12^	0.225^12^
NGC 6026	*TESS*	2458649.84959+0.5283282$\times$E	0.20	Ell^45^	0.53^41^, 0.53^45^

Table 3 summarises the properties of the Group II objects. The newly derived periods are in excellent agreement with published values for all systems except one, demonstrating the power and reliability of *Gaia* for this type of analysis. For the CS of PPA J1747-3435, we determine a period of 0.44 d, approximately twice the 0.2247 d reported by^42^. The *Gaia* light curve clearly reveals a primary eclipse, supporting an eclipsing binary interpretation rather than the previously suggested irradiated binary scenario. Therefore, it is important to note the possible misclassifications between ellipsoidal modulation and irradiation effect. In close binary systems, ellipsoidal modulation produces two similar maxima per orbit, which can be mistaken for irradiation with half the true period. Conversely, asymmetric irradiation patterns may mimic ellipsoidal curves. Our classifications are based on the morphology of phase-folded light curves, but spectroscopic follow-up is needed to confirm orbital periods and distinguish between these effects. We encourage caution in interpreting the variability types listed in Tables 2 and 3, especially for low-amplitude systems.

**Fig. 4:**
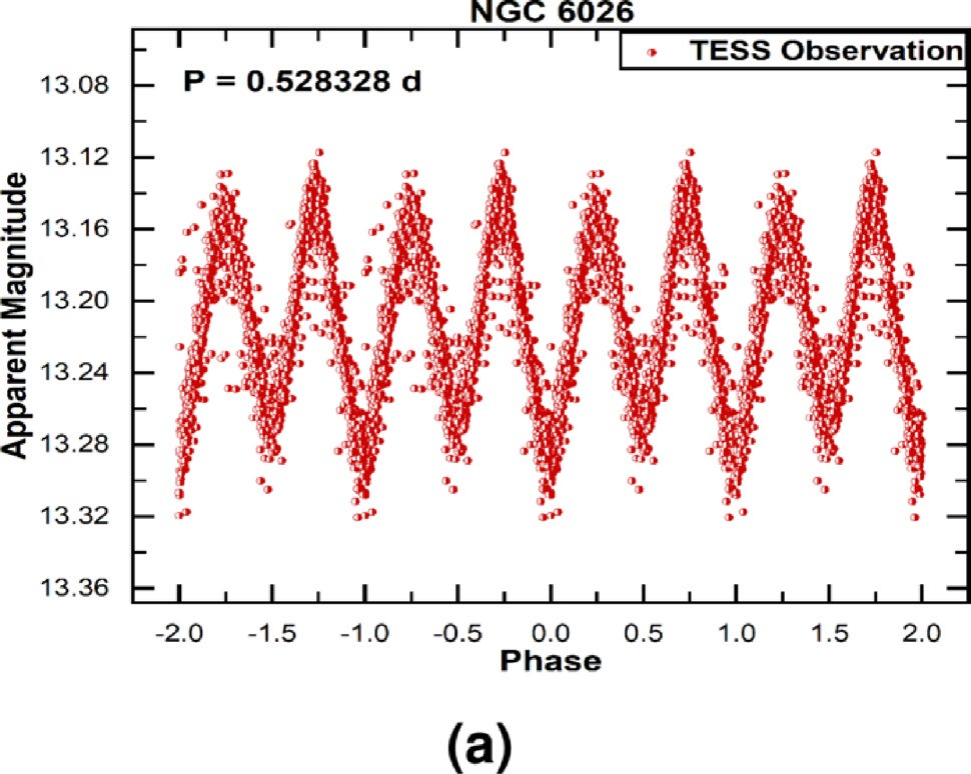
The phase-folded light curve NGC 6026, created from *TESS* photometric data.

The light curves of Group II objects, shown in Fig. 3, emphasise the effectiveness of *Gaia* time-domain photometry in detecting and characterising binary CSPNe. Figure 4 presents the phase-folded light curve of NGC 6026 from *TESS* photometric data.

### Binary fraction

One primary aim in researching PNe evolution is to determine what fraction of CSs belong to binary systems–especially those with close orbits that allowed substantial interaction during nebula formation. This study offers a chance to measure the binary fraction in a well-characterized set of CSPNe and to contrast it with earlier estimates. After excluding two objects now classified as chance alignments (PN M3-2 and PN SuWt 2), the final sample used for the binary-fraction analysis consists of 79 CSPNe. Within this group, we identified 32 previously known close binary central stars and 6 newly detected ones, for a total of 38 close binaries. This corresponds to a short-period binary fraction of 48%. This fraction is signicantly higher than the reported value of 10–15%^43^, 12–33%^44^, 12–21%^12^, 20.7–23.5.7.5%^16^. The apparent discrepancy does not imply a contradiction; it arises because our analysis was based on a preselected sample of Gaia DR3 variable CSPNe^18^, whereas previous studies typically derived fractions with respect to the entire population of observed PNe, encompassing both variable and non-variable objects.

## Conclusions

By combining *Gaia* DR3 photometry with complementary data from *TESS*, *OGLE*, and *Kepler*, our analysis of 81 CSPNe has substantially increased the number of confirmed binary central stars, adding 17 new variable systems and validating 15 previously identified binaries. The results demonstrate the effectiveness of *Gaia* DR3 time-series photometry in detecting the low-amplitude variability typical of close binaries, enabling both confirmation of known systems and discovery of new ones. A key outcome is the strong morphological link between close binarity and nebular structure: nearly 80% of nebulae hosting the newly confirmed short-period binaries display bipolar or elliptical morphologies. This supports the view that binary interactions are central to the origin of asymmetry in PNe. Cases of apparently round nebulae can likely be explained by moderate viewing inclinations or projection effects, rather than strictly pole-on orientations, ensuring consistency with observed radial-velocity variations where available. We derive a short-period binary fraction of 48% for our Gaia DR3 variability-selected sample, a value that is higher than earlier estimates derived from non-selected samples, emphasizing how sample selection affects binary fraction calculations. Our analysis also highlights the distinction between close and wide binaries: while wide binaries show a bipolar/elliptical fraction of 57–70%, this remains lower than the fraction observed for close binaries, underscoring the stronger shaping influence of close interactions. The forthcoming *Gaia* DR4, with its longer baseline (66 months versus 34 in DR3), is expected to deliver higher precision and reveal an even larger and more diverse binary population. Overall, this work reinforces the fundamental role of binary interactions in PN shaping, while showcasing the capacity of *Gaia* ’s all-sky photometric survey –especially when combined with other time-domain missions–to drive progress in this area of research.

## Data Availability

The data underlying this article are publicly available through the following archives: Gaia Data Release 3 via the ESA Gaia archive (https://gea.esac.esa.int/archive/), TESS and Kepler data via the Mikulski Archive for Space Telescopes (MAST) (https://mast.stsci.edu/), and OGLE photometry via the OGLE project homepage (https://ogledb.astrouw.edu.pl/ogle/OCVS/catalog_query.php).
