# Correction: Exploring central star variability of planetary nebulae using gaia photometry

**DOI:** 10.1038/s41598-026-64053-2

**Published:** 2026-07-24

**Authors:** M. A. NegmEldin, A. Ali, G. M. Hamed, A. Essam

**Affiliations:** 1 Astronomy, Space Science & Meteorology Department, Faculty of Science, Cairo University, Giza, 12613 Egypt; 2 Stellar Astronomy Lab, Astronomy Department, National Research Institute of Astronomy and Geophysics, 11421 Helwan, Cairo, Egypt

Correction to: *Scientific Reports* 10.1038/s41598-026-42163-1, published online 25 March 2026

The original version of this Article contained error in the name of the author G. M. Hamed, which was incorrectly given as G. M. Hamid.

The original Article has been corrected.

